# Impact of pilot diesel injection timing on performance and emission characteristics of marine natural gas/diesel dual-fuel engine

**DOI:** 10.1038/s41598-024-61672-5

**Published:** 2024-05-10

**Authors:** Xiao Zhang, Jianqun Gao, Dawei Fan, Qizheng Yang, Fangjun Han, Hongliang Yu

**Affiliations:** 1 School of Ocean, Yantai University, No. 30, Qingquan Road, Laishan District, Yantai, 264005 China; 2 Yantai CIMC Raffles Offshore Limited, 70 Zhifu Island East Road, Yantai, 264000 China; 3 Dalian Scientific Test and Control Technology Institute, 760 Research Institute, China Shipbuilding Industry Group Co., Ltd, 14 Binhai Street, Dalian, 116026 China

**Keywords:** Combustion stage, Dual fuel engine, Flame spread velocity, Pilot diesel injection timing, Mechanical engineering, Diesel fuel, Natural gas

## Abstract

In diesel-ignited natural gas marine dual-fuel engines, the pilot diesel injection timing (PDIT) determines the premixing time and ignition moment of the combustible mixture in the cylinder. The PDIT plays a crucial role in the subsequent development of natural gas flame combustion. In this paper, four PDITs (− 8 °CA, − 6 °CA, − 4 °CA, and − 2 °CA) were studied. The results show that the advancement of PDIT increased the engine's power, thermal efficiency, and natural gas flame spread velocity, and increased NO emissions and CH_4_ emissions of the marine engine. The PDIT affected the ignition delay period and the rapid combustion period to a greater extent than the slow combustion period and the post combustion period. With each 2 °CA advancement of PDIT, the engine's power increased by 69.87 kW, thermal efficiency increased by 0.42%, radial flame spread velocity increased by 2 m/s, axial flame spread velocity increased by 1.7 m/s, NO emissions increased by 6.1%, and CH_4_ emissions increased by 3.75%.

## Introduction

Green and low-carbon development has become a global consensus. The global trend of using ships for transportation has continued to expand in recent years^1^, and the transportation sector is an important battlefield for the realization of the “carbon peaking and carbon neutrality” strategy, and the shipping industry plays an important role in this process. The 78th session of the International Maritime Organization's Marine Environment Protection Committee (MEPC 78), held remotely in June 2022, highlighted new mandatory measures to reduce the carbon intensity of international shipping to achieve a sustainable shipping industry by 2050^2^. The goal of reduces greenhouse gas emissions by 50% from 2008 levels. To build a clean, low-carbon, safe and efficient energy system, liquefied natural gas (LNG), as a clean and efficient low-carbon fossil energy, has attracted more and more attention in the field of marine engines^3^. In recent years, the number of marine engines using natural gas as the green and low-carbon alternative fuel has increased rapidly.

Natural gas/diesel dual-fuel (NDDF) engine technology is an important aspect for decreasing greenhouse gas (GHG) emissions of marine engines. Many scholars had done experimental research on natural gas engine^4–10^. The experimental research method is suitable for small and medium-sized engines. For large-scale low-speed dual-fuel marine engines, the research method of data collection through hundreds or thousands of tests has space and equipment limitations. Numerical research on natural gas engine^11–16^ were mainly concerned with the impact on the parameters. Many parameters such as swirl ratio^17^, exhaust gas recirculation (EGR)^18^, premixed ratio^19^, injection strategy^20,21^, spray angle^22,23^, fuel characteristics^24–26^, piston bowl geometry^27,28^, and start of injection timing^29^ affected the RCCI engine’s performance and emission^30^. Marine low-speed two-stroke dual-fuel engines have different scavenging styles, injector positions, and combustion chamber styles than small and medium-sized engines. The emission laws for marine two-stroke machines cannot directly use the laws for four-stroke engines. Researchers had extensively explored the optimization methods of combustion and emission performance of natural gas engines^31–34^, but most research focus on the impact of operating conditions on performance^35–39^, and few studies were conducted on the quantification of combustion process and the control of combustion stages for marine large-bore engines.

In a direct injection natural gas engine, pilot diesel injection timing (PDIT) has a significant impact on the ignition timing and combustion quality of the combustible mixture in the cylinder. In a dual-fuel engine ignited by diesel, PDIT directly affects the ignition delay period (IDP) of the engine and plays a crucial role in whether the natural gas and air in the cylinder can be well mixed. Too much advance or too much lag of PDIT will also lead to too low cycle thermal efficiency of the engine. Poor PDIT cannot ensure efficient combustion and affect the working ability of the engine. Therefore, the computational fluid dynamics simulation technology and image quantitative research method were used to conduct a detailed numerical study on the low-speed two-stroke NDDF engine under different PDIT. In the study, the PDIT was changed, but the other boundary conditions such as initial state, load, speed, and natural gas injection timing (NGIT) were kept unchanged. The PDIT change was two crank angle deg (°CA), and the change range was from − 8 to − 2 °CA.

## Methodology

### Engine bench test

MAN B&W 6S50ME-C-GI engine is an NDDF engine with in-cylinder high-pressure injection. The engine is presently used as the main engine of offshore barges, marine police enforcement vessels, ocean-going container vessels and LNG vessels. Main data of engine are shown in Table 1.

**Table 1:** Main data of engine.

Name	Data	Name	Data
Cylinder number	6	Engine type	2-stroke
Bore × Stroke/mm	500 × 2000	Method of aspiration	Pressure charged
Rated speed/(r/min)	108	Rated power/kW	8100
Diesel injection holes	5 × *Φ*1.05 mm	NG injection holes	4 × *Φ*2.2 mm
Maximum cylinder pressure/MPa	17	Nominal compression ratio	15

**Figure 1:**
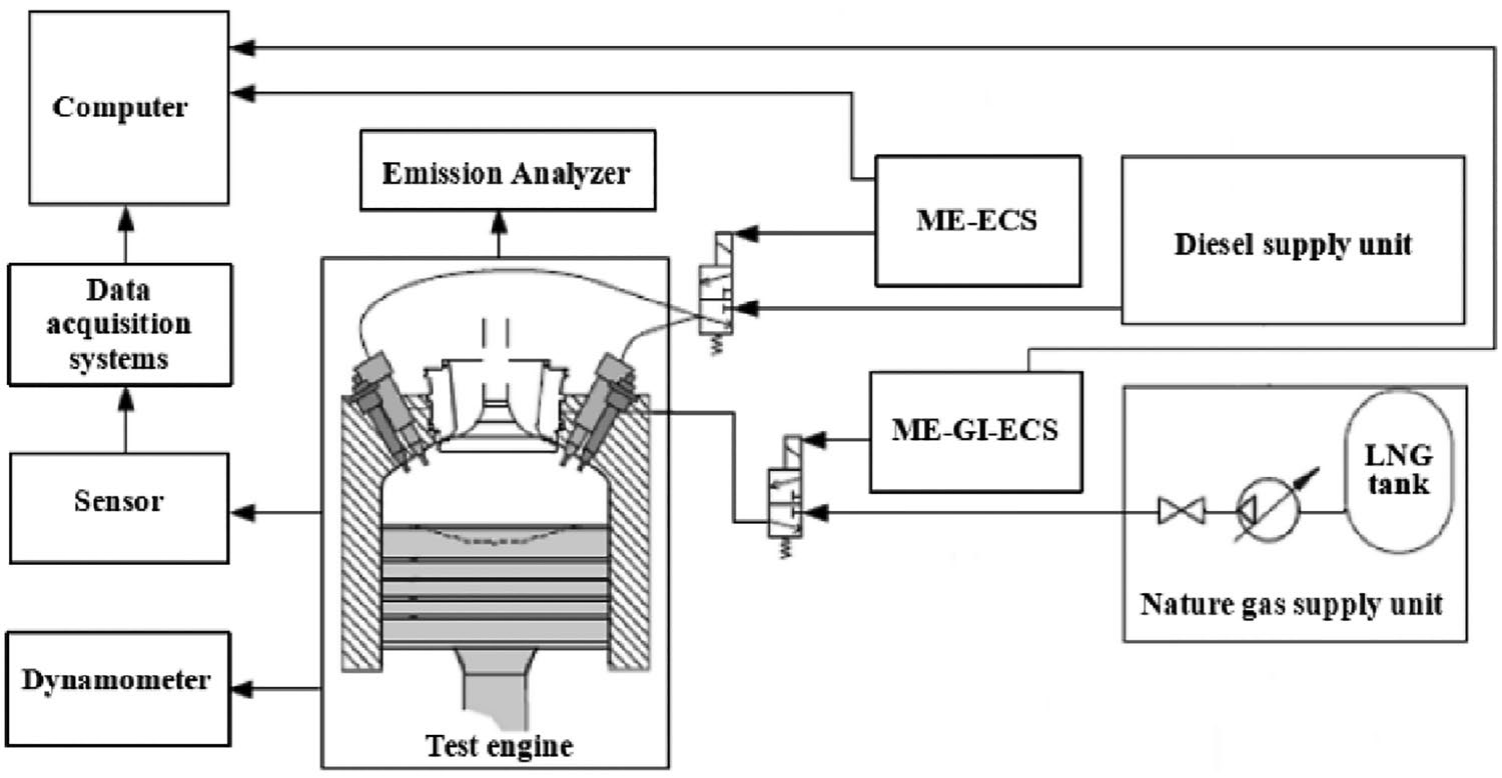
Bench test.

The marine engine factory conducted bench tests of propulsion characteristics before the main engine left the factory to ensure that the main engine operated safely and stably, and can be reliably remote controlled and operated. All indicators of marine engines meet the requirements of ship inspection authorities. The schematic diagram of the bench test is shown in Fig. 1. The diesel particulate emission collection equipment uses HOR1BAMDLT-1302TMA, the exhaust analyzer uses HORJBAMEXA-1600DS, and the other test equipment is shown in Table 2.

**Table 2:** Test main instruments.

Analyzer	Model	Measurement ranges	Deviation (%)
CO/10^–6^	AIA 240	0–1000	0.16
CO_2_/%	AIA 240	0–16.0	0.12
NO/10^–6^	FAC 246	0–2000	0.11
O_2_/%	IMA 241	0–25	− 0.10
HC/10^–6^	FAC 246	0–2000	0.15
*t*/^o^C	FC2022	0–1000	0.1
*p*/kPa	FC2022	− 50 to 4000	0.1
speed/(r/min)	FC 2010	0–3000	0.1
Torque/N m	CFSR-26	0–37,300	0.25
Fuel flow/(kg/h)	FC 2210	0–1000	0.1
LNG flow/(m^3^/h)	CMF 200 M	0–2000	0.2

All equipment must be calibrated before the test to ensure its effectiveness. The test focuses on marine dual-fuel engines and their propulsion characteristics. The engine runs for 30 min at a power of 2025 kW and a speed of 68 revolutions per minute (rpm). After stable operation at 4050 kW power and 85.7 r/min speed, 6075 kW power and 98.1 r/min speed, and 8100 kW power and 108 r/min speed, the operational and emission data were measured for each respective load condition.

### Calculation model

**Figure 2:**
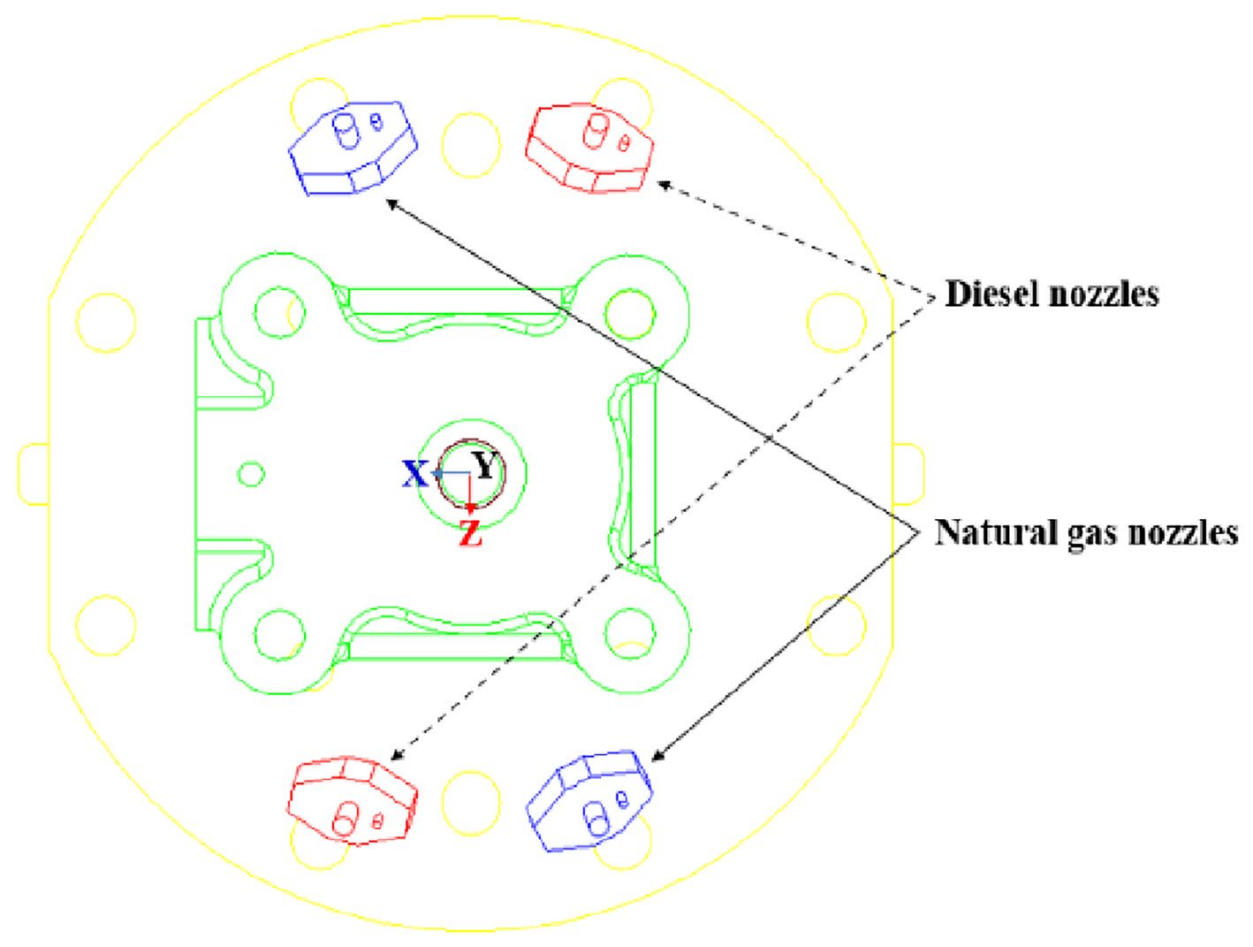
Nozzles arrangement on cylinder cover.

**Figure 3:**
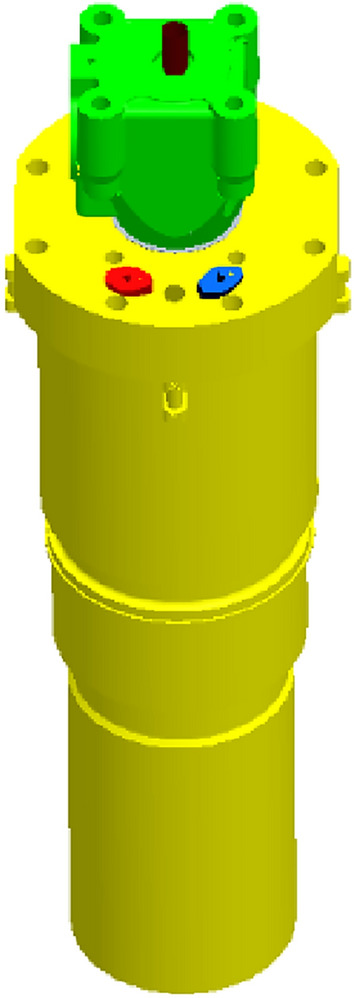
Combustion chamber assembly.

**Figure 4:**
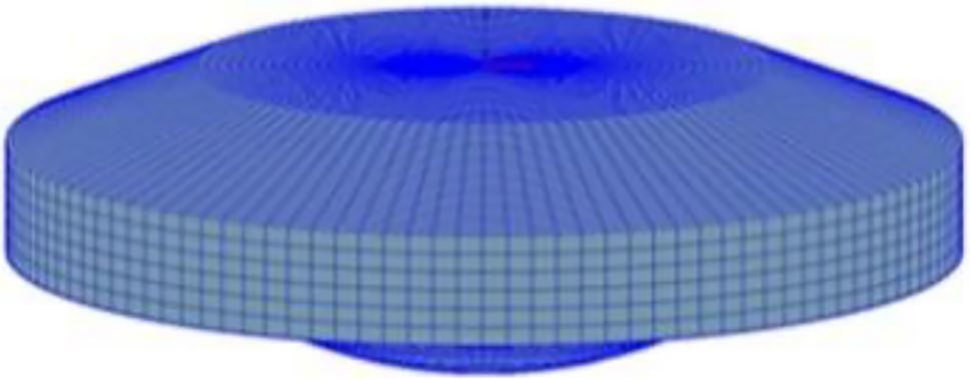
The mesh calculation model of combustion chamber .

For marine dual-fuel engines with cylinder diameters less than 500 mm, two natural gas and two diesel nozzles were set for each cylinder. The nozzles arrangement is shown in Fig. 2. The nozzle extended 35 mm deeply into the cylinder. Diesel nozzles had five holes, holes towards the cylinder side of the 63° angle range. The natural gas nozzle had four holes, and the holes were oriented toward the cylinder center at a 51° angle range. The volume of the combustion chamber was measured using a three-dimensional (3D) assembly model (Fig. 3). The established combustion chamber model was then imported into AVL FIRE Version 8 (https://www.avl.com) software to create the computational volume grid using the Hybird Assistant module in Fame Meshing. Finally, the transient moving grid was divided by the Fame Engine module in Fame Motion. Due to the large size of the engine combustion chamber and the large number of static volume grids generated, the FIRE software imposes strict requirements on the quality of the moving grid in the numerical calculation of the combustion process. During the transient moving grid division process from the volume grid, irregular grid or grid deformation often occurs, leading to non-convergence, divergence, and abnormal termination of the calculation process. After numerous attempts and mesh independence calculations with various grid sizes, it was discovered that dividing the moving grid into 2 cm unit lengths and the non-moving grid into 1 cm unit lengths significantly enhances calculation speed and accuracy. The mesh calculation model of the combustion chamber is shown in Fig. 4.

### Condition terms

We used the 3D Computational Fluid Dynamics (CFD) software AVL FIRE to calculate the in-cylinder flow, spray, and combustion process of the engine. The top dead center (TDC) is 0 °CA. The combustion calculation started at 138 °CA before the top dead center (BTDC) at the moment of scavenging port closing (SPC) and ended at 114 °CA after the top dead center (ATDC) at the moment of exhaust valve opening (EVO). The initial pressure in the cylinder was 3.34 bar, the initial temperature was 372 K, and the gas composition was set according to the scavenging air. The calculated data at 8100 kW power and 108 r/min speed were given in Table 3. The in-cylinder flow field was calculated using a k − ε turbulence model^40^. The fuel spray, breaking, fragmentation, and evaporation processes were calculated using Walljet1, Wave, and Multicomponent models^41,42^. The transmission, ignition, and combustion processes of each component were calculated by the Coherent Flame combustion model^43,44^. The emission model commonly used was the Heywood original NOx model and the Kinetic soot emission model^45^.

**Table 3:** Calculated data.

Name	Data	Name	Data
NGIT/°CA	− 4 to 20	NG temperature/°C	45
NG injection pressure/MPa	30	NG supply/(g/r)	182.94
PDIT/°CA	− 8 to − 4, − 6 to − 2, − 4 to 0, − 2 to 2	Diesel temperature/°C	38
Diesel injection pressure/MPa	35	Diesel supply/(g/r)	7.26
EVO/°CA	114	SPC/°CA	− 138
Valve temperature/°C	570	Piston temperature/°C	388
Liner temperature/°C	218	Pilot diesel ratio/%	5

### Validation of numerical modeling

**Figure 5:**
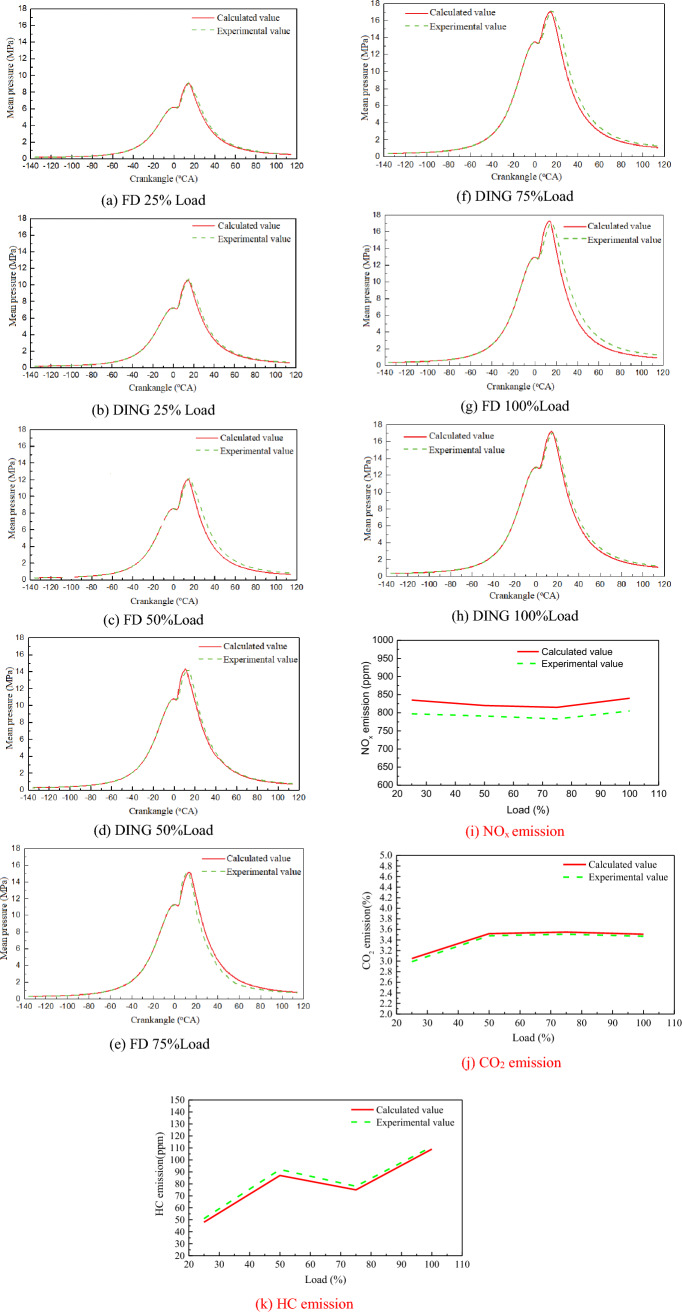
Comparison between calculated value and experimental value.

The test was carried out on the engine test cell of the marine diesel engine factory. Figure 5 shows the comparison of indicator diagram pressures of the engine under 25%, 50%, 75%, and 100% load. Comparing the pressure curves of each load, it was found that there was a certain deviation between the calculated values and the experimental values. The reasons are analyzed as follows: First, natural gas was set to CH_4_ with 100% in the calculation under the diesel ignited natural gas (DING) mode. Second, the diesel reaction mechanism in full diesel (FD) mode was set as an n-heptane reaction mechanism. Third, the air involved in the numerical combustion calculation was set as the ideal gas. Although there were deviations, the overall linear trend of the calculated value was consistent with the experimental values, which showed that the calculation model could correctly reflect the characteristics of the combustion process of the engine. The calculated value of CO_2_, NOx and HC were agreement well with the experimental value in the overall linear trend, indicating that the emission model could accurately predict the emission characteristics of DING combustion conditions.

## Results and discussion

### Mean pressure and temperature analysis

**Figure 6:**
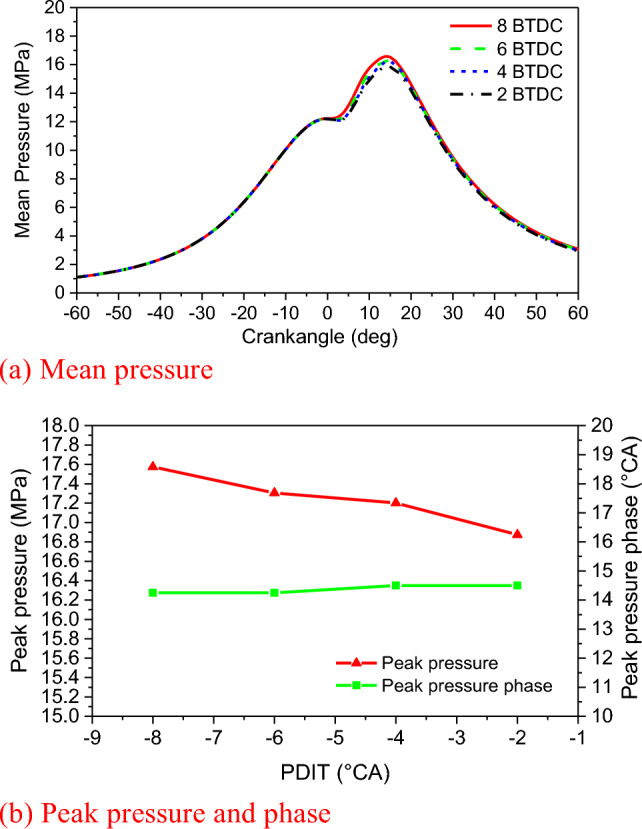
Impact of PDIT on pressure and phase.

**Figure 7:**
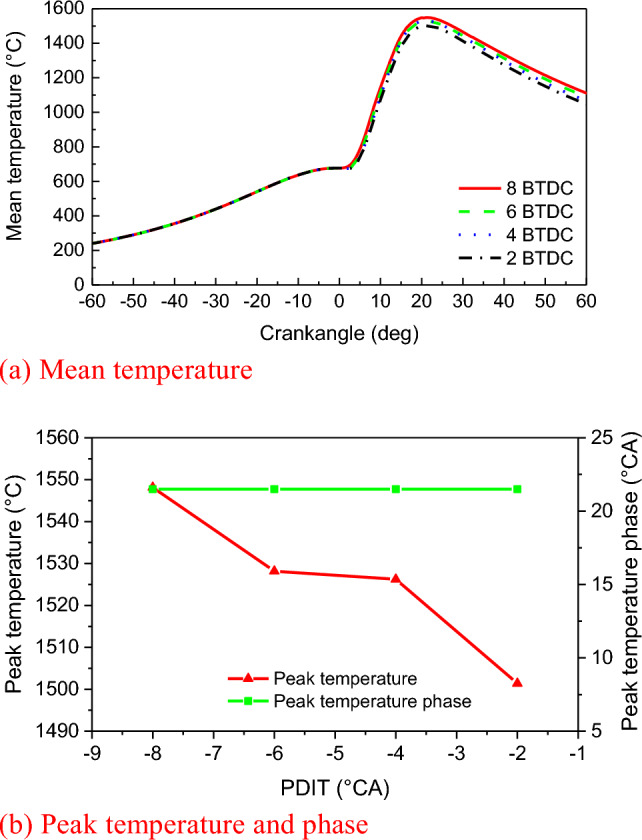
Impact of PDIT on the temperature and phase.

The engine speed is 108 r/min at 100% load, with a total fuel mass of 31.7 g in a single-cylinder, single-cycle combustion chamber. This includes 30.49 g of natural gas and 1.21 g of diesel fuel. Figure 6 and Fig. 7 show that the in-cylinder mean pressure and in-cylinder mean temperature curves also show two peaks. The reasons are consistent with those in the NGIT study^43^. The maximum in-cylinder burst pressure gradually advances and increases with the advancement of PDIT in the Fig. 6. For every 2 °CA advance of PDIT, the maximum in-cylinder burst pressure increases by 1.4%. The highest combustion pressure of 17.57 MPa occurs at − 8 °CA of PDIT. At this point, the engine does not experience knocking because both natural gas and diesel undergo high-pressure direct injection before reaching top dead center. The diesel fuel ignites first, preparing for the subsequent ignition of the injected natural gas. Natural gas is injected into the cylinder at − 4 °CA BTDC, primarily for the diffusion combustion of the Diesel cycle, which explains the engine's low tendency for knocking. The advancement of PDIT causes the ignition moment to advance and IDP to be short, resulting in a rapid increase in pressure during the rapid combustion period (RCP), the combustion phase moving to the TDC, and the combustion phase continuously shifting forward, increasing the engine's work capacity. Therefore, if the PDIT is reasonably advanced, the combustion will be completed near the TDC, the combustion will be more adequate and the engine will be more powerful. During the PDIT process from − 2 to − 8 °CA, the engine power increased by 210 kW, and the thermal efficiency increased by 1.27%. Calculated based on the lower heating value of natural gas at 48,280 kJ/kg, this is equivalent to saving 4.74 g of natural gas per cycle. With each 2 °CA advancement of PDIT, the engine's power increased by 69.87 kW, thermal efficiency increased by 0.42%. In Fig. 7, as the PDIT is advanced, the maximum in-cylinder temperature also appears to advance and increase. For every 2 °CA of PDIT advance, the maximum in-cylinder temperature increases by 0.5%, while the peak in-cylinder temperature phase almost does not change, which is because the diesel fuel ignited with natural gas only accounts for 5% of the total calorific value, and has little impact on the peak temperature in the cylinder.

### Combustion period analysis

**Figure 8:**
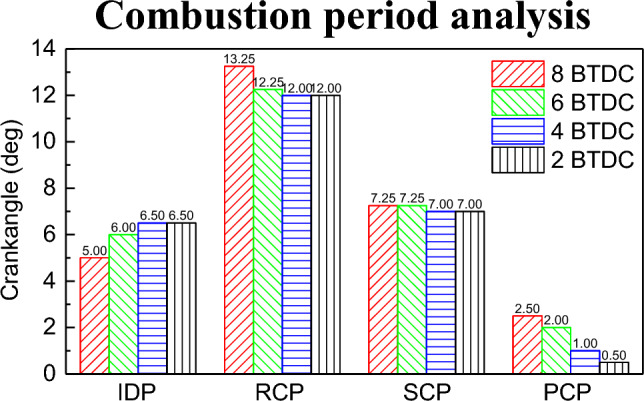
Impact of PDIT on combustion period.

Figure 8 shows the impact of PDIT on each stage of the in-cylinder combustion process. Figure 8 shows that the PDIT is constantly advanced, the IDP becomes shorter, and the RCP, slow combustion period (SCP), and the post combustion period (PCP) become longer. Early PDIT causes ignition moment to be earlier, the IDP becomes shorter, leading to insufficient premixing of NG and air. Premature ignition causes the temperature inside the cylinder to rise prematurely. This leads to an increase in the diffusion rate of the natural gas injected later due to the elevated temperature in the cylinder. Natural gas forms a combustible mixture with air when diffused. Additionally, the early ignition of diesel provides energy, accelerating the combustion velocity of natural gas and prolonging the RCP. The SCP and PCP become longer causing a large amount of CH_4_ to stall in the cylinder.

### High temperature volume analysis

**Figure 9:**
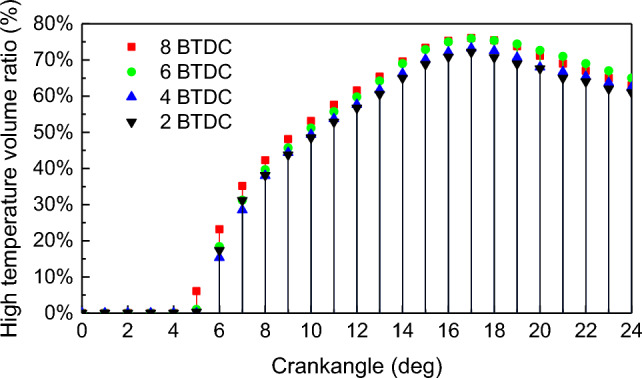
Impact of PDIT on high temperature volume ratio.

**Figure 10:**
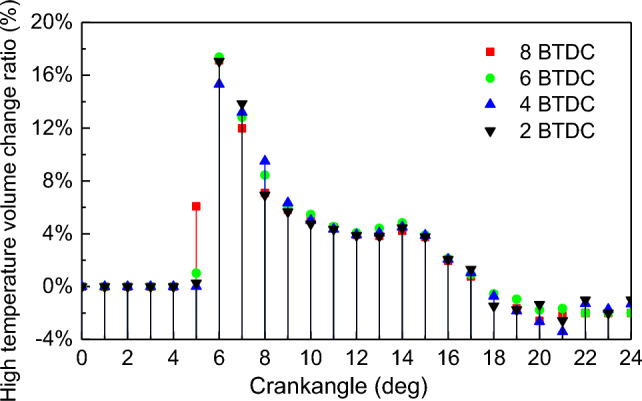
Impact of PDIT on high temperature volume change ratio.

To investigate the frequent occurrence of combustion instability in natural gas/diesel dual-fuel engines, an analysis was conducted on the volume of the region inside the cylinder where the temperature exceeds 1800 K during the combustion process. Due to the transient nature of natural gas combustion in the cylinder of dual-fuel engines, the stability of the combustion process is primarily indicated by the fluctuation in the volume of the high-temperature flame region. Figures 9 and 10 show that the volume ratio and volume change ratio of high temperature at different PDIT show the same law as that under the NGIT: both of them are “increasing and then decreasing” of law^43^. The maximum high temperature volume ratio was 72.2%, 73.3%, 75.9%, and 76.1% under the PDIT on − 2 °CA, − 4 °CA, − 6 °CA and − 8 °CA. The maximum high temperature volume ratio rise by 1.3% for PDIT advanced every 2 °CA. The law is also consistent with that under NGIT: the advancement of PDIT leads to a larger high temperature volume in-cylinder. With the PDIT from − 2 to − 8 °CA, the maximum high temperature volume change ratio is 17.1%, 15.3%, 17.4%, and 17.2%, respectively. The high temperature volume change ratio rise by 0.03% for PDIT advanced every 2 °CA. Based on the linear pattern of the high-temperature volume change ratio curve in Fig. 10, it can be concluded that unstable combustion mainly occurs at 5–9 °CA. To maintain stable engine combustion, PDIT should be appropriately delayed.

### Flame spread velocity analysis

The high-temperature flame spread velocity in the cylinder can be calculated by measuring the distance the high-temperature flame boundary moves within a unit of time. By capturing instantaneous images within the cylinder for a specific time interval, segmenting the high-temperature flame boundary in image processing, and identifying the displacement coordinates of the flame boundary points A (x_i_, y_i_), it is possible to calculate the distance(∆l) of consecutive movements of the boundary points.

1 $$ \Delta l = \sqrt {\left( {x_{i + 1} - x_{i} } \right)^{2} + \left( {y_{i + 1} - y_{i} } \right)^{2} } $$

2 $$ v = \Delta l/\Delta t $$

*∆l* is the distance of consecutive movements of the boundary points. *v* is the flame propagation velocity. *∆t* is the time interval between two consecutive movements of the flame boundary point A.

**Figure 11:**
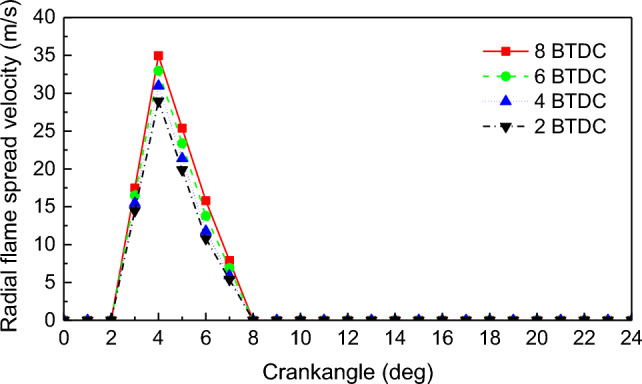
Impact of PDIT on the radial flame spread velocity.

**Figure 12:**
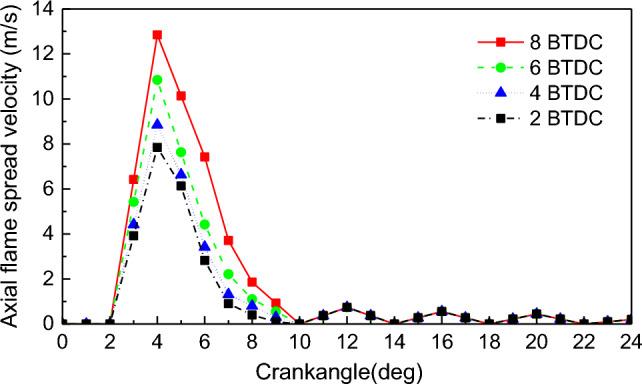
Impact of PDIT on the axial flame spread velocity.

Figures 11 and 12 show the radial flame spread velocity and axial flame spread velocity in-cylinder under different PDIT, respectively. The linear law of flame spread velocity under different PDIT is consistent with that under NGIT: the radial flame spread velocity has a bump fluctuation, and the axial flame spread velocity has a wavy fluctuation. With the PDIT changed from − 2 to − 8 °CA, the maximum radial flame spread velocity changed from 28.9 to 34.9 m/s, and the maximum axial flame spread velocity changed from 7.8 to 12.8 m/s. The maximum radial and axial flame spread velocity rise by 2 m/s and 1.7 m/s for the PDIT advanced every 2 °CA.

### CH_4_ combustion interruption analysis

**Figure 13:**
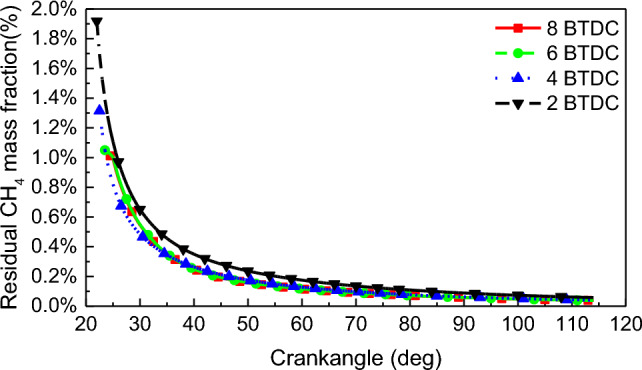
Residual CH_4_ mass fraction after the end of combustion.

Figure 13 shows the residual CH_4_ mass fractions in-cylinder after combustion completes at different PDIT. The advancement of PDIT caused the reduction of residual CH_4_ in the cylinder after combustion completes. The residual CH_4_ in-cylinder of combustion completes at PDIT of − 2 °CA, − 4 °CA, − 6 °CA, and − 8 °CA, the mass fractions were 1.92%, 1.31%, 1.05%,and 1.01%, respectively. Residual CH_4_ mass fractions at each PDIT with the EVO were 0.038%, 0.040%, 0.046%, and 0.058%, respectively.

**Figure 14:**
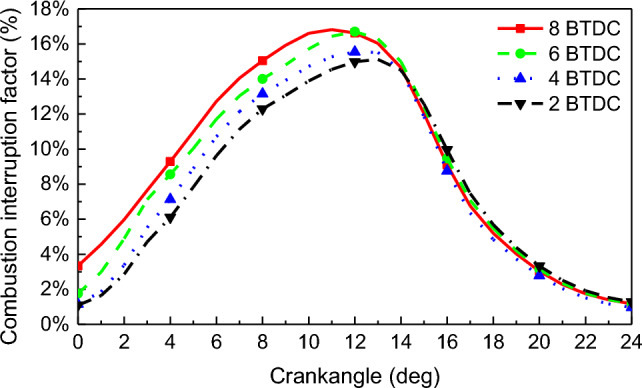
Combustion interruption factor.

The main reason for the high CH_4_ emissions in dual-fuel engines is incomplete combustion in the cylinder. The incomplete combustion of CH_4_ is mainly due to the CH_4_ concentration inside the cylinder not reaching the ignition limit and the local temperature inside the cylinder being too low for CH_4_ to ignite temperature (650 °C). Therefore, the phenomenon where the CH_4_ flame cannot propagate further due to the above two reasons is called combustion interruption. The region where the temperature in the combustion chamber is below 650 °C and the residual CH_4_ mass fraction exceeds 0 is termed as the incomplete combustion zone of CH_4_. The ratio of the incomplete combustion area of CH_4_ to the full combustion chamber area is defined as the combustion interruption factor. Figure 14 shows that with the advance of PDIT, the combustion interruption factor gradually moves forward, the peak of the combustion interruption factor gradually rises, and the peak of the combustion interruption factor gradually increases from 15.12% at − 2 °CA to 16.81% at − 8 °CA. In the case of constant NGIT, the NG injected into the cylinder met the ignition flame, and the combustion ratio of NG became faster due to the advancement of PDIT to make the ignition energy in-cylinder sufficient. It made CH_4_ and air mix badly and caused the combustion interruption to increase. The combustion interruption factor rise by 0.57% for PDIT advanced every 2 °CA.

### Emissions analysis

**Figure 15:**
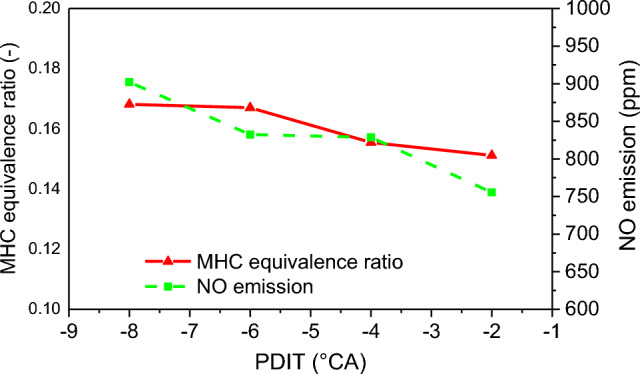
MHC equivalence ratio and NO emission under different PDIT.

The fuel conditions of dual-fuel engines are divided into pure diesel conditions and diesel-ignited natural gas conditions. To distinguish between the hydrocarbon emissions in the diesel-ignited natural gas condition and the pure diesel combustion condition of the engine, the hydrocarbon emissions, mainly unburned methane, in the diesel-ignited natural gas condition are denoted as methane-based unburned hydrocarbon (MHC). Figure 15 shows the law of NO emission and MHC equivalent ratio under different PDIT. The MHC ratio gradually decreases as the PDIT moves toward TDC, and the decrease rate increases between − 6 and − 4 °CA. The NO emission shows a decreasing trend with the delay of PDIT. The MHC equivalent ratio decreases by 3.75% for each 2 °CA delay of PDIT. The NO emission decreases by 6.1% for each 2 °CA delay of PDIT.

## Conclusion

The PDIT has an extremely important influence on the NG mixture and the effective combustion organization. The impacts of PDIT on flame spread velocity, high temperature volume, CH_4_ combustion interruption, and emission products of marine low-speed two-stroke dual-fuel engine combustion process had investigated when PDIT varies from − 2 to − 8 °CA, respectively.

The advancement of PDIT accelerates the flame combustion velocity of in-cylinder NG, shifts the combustion phase toward TDC, makes the exothermic process more concentrated. With each 2 °CA advancement of PDIT, the engine’s power increased by 69.87 kW, thermal efficiency increased by 0.42%. The unstable combustion mainly occurs at 5–9 °CA. To maintain stable engine combustion, PDIT should be appropriately delayed.

The PDIT affects the IDP and RCP to a greater extent than the SCP and PCP. Marine NDDF engines can control the IDP and RCP by changing the PDIT. Delaying the PDIT can reduce the high temperature volume in the cylinder, which can reduce the combustion interruption factor of NG, and reduce NO emission and CH_4_ emission. The proper delay of PDIT is beneficial to the marine NDDF engine to achieve carbon peak and carbon neutrality.

With the PDIT advanced every 2 °CA, the mean pressure and mean temperature rise by 1.4% and 0.5%, the high temperature volume and its change ratio rise by 1.3% and 0.03%, the radial and axial flame spread velocity accelerate by 2 m/s and 1.7 m/s, NO emission rises by 6.1%, combustion interruption factor rises by 0.57%, and MHC emission rises by 3.75%.

## Data Availability

The data that support the findings of this study are available from Yantai CIMC Raffles Offshore Limited but restrictions apply to the availability of these data, which were used under license for the current study, and so are not publicly available. Data are however available from the authors upon reasonable request and with permission of Yantai CIMC Raffles Offshore Limited. Please contact Hongliang Yu if you need data from this study.
